# Breeding system and spatial isolation from congeners strongly constrain seed set in an insect-pollinated apomictic tree: *Sorbus subcuneata* (Rosaceae)

**DOI:** 10.1038/srep45122

**Published:** 2017-03-24

**Authors:** Tracey J. Hamston, Robert J. Wilson, Natasha de Vere, Tim C. G. Rich, Jamie R. Stevens, James E. Cresswell

**Affiliations:** 1 Biosciences, University of Exeter, Exeter, Devon, UK; 2 Whitley Wildlife Conservation Trust, Paignton Zoo, Paignton, UK; 3 National Botanic Garden of Wales, Llanarthne, UK; 4 Institute of Biological Environmental and Rural Sciences, Aberystwyth University, Aberystwyth, UK; 5 57, Aberdulais Road, Cardiff, UK

## Abstract

In plants, apomixis results in the production of clonal offspring via seed and can provide reproductive assurance for isolated individuals. However, many apomicts require pollination to develop functional endosperm for successful seed set (pseudogamy) and therefore risk pollination-limitation, particularly in self-incompatible species that require heterospecific pollen. We used microsatellite paternity analysis and hand pollinations to investigate pollen-limitation in *Sorbus subcuneata*, a threatened endemic tree that co-occurs with its congener, *S. admonitor*. We confirmed that *S. subcuneata* is an obligate pseudogamous apomict, but open-pollinated flowers rarely produced seed (flower-to-seed conversion < 1%) even though they rapidly accumulated pollen on their stigmas. Manual heterospecific pollination by *S. admonitor* resulted in a high flower-to-seed conversion rate (65%), however, we estimate that the ratio of self: heterospecific pollination in open-pollinated flowers was at least 22:1. Despite the efficacy of heterospecific pollination, the contribution of *S. admonitor* trees to paternity in seed from open-pollinated flowers of *S. subcuneata* decreased rapidly with the spatial separation between paternal and maternal trees. Conservation efforts aimed at maintaining species with this breeding system must therefore manage the congeners in tandem which will also maintain the potential for rare heterospecific fertilisation that typically cause rapid diversification in these lineages.

Asexual reproduction through clonal seed (apomixis) offers reproductive assurance to isolated individuals^1^ and is often associated with colonising species that have wider geographic distributions than their sexual counterparts^2^. Apomixis is widespread among angiosperm families^3^, but occurs more frequently in the Asteraceae, Poaceae and Rosaceae^4^,^5^, where it is associated with polyploidy^6^. Many apomicts are also pseudogamous, which means that they require pollen to develop functional endosperm for the maturation of their otherwise clonal seed. For species with a pseudogamous apomictic (PA) breeding system, the requirement for pollination may limit seed set just as it often does in sexual species^7^.

In the Rosaceae, many polyploid pseudogamous apomicts are self-compatible^8^, which enables seed set through autogamous (within flower) or geitonogamous (within individual) pollen transfer. Curiously, however, some PA species in the Rosaceae are self-incompatible (SI)^9^, which means that they require heterospecific pollination by congeners. SI-PA species possess a breeding system that seemingly exposes them to the pressures of pollen-limitation without conferring any obvious adaptive benefits. We investigated the performance of this perplexing breeding system in a woodland community of a rare member of the Rosaceae, a putatively self-incompatible triploid *Sorbus* species.

Pollen-limited seed set can have various causes related to the quantity and quality of pollen available to females^10^. Factors that limit the quantity of pollen delivered to stigmas include flowering asynchrony between males and females and inadequate service from pollen vectors (insects or wind). Pollen quality limits seed set when too much of the pollen that reaches a female’s stigmas is incompatible. SI-PA species are therefore vulnerable to seed limitation by both the quality and quantity of pollen available to females caused by spatial isolation from compatible congeners. The degree of limitation can be measured by the increase in seed produced when pollen is added to the stigma by hand^11^, but the ecology of pollen-limited seed set in PA species has rarely been investigated.

The focal species for the present study is a member of the genus *Sorbus* L., which comprises small and medium-sized trees that produce corymbs of showy, hermaphrodite flowers during late spring and early summer. In common with many other members of the Rosaceae^12^,^13^, the floral architecture in *Sorbus* is entomophilous and its flowers attract generalist flower-visiting insects, mainly bees^14^. Diploid *Sorbus* species are typically self-incompatible out-crossers^13^,^15^, but *Sorbus* also contains apomictic polyploids derived from hybridisation^3^,^16^. Recent speciation is evident in *Sorbus*^17^,^18^,^19^ and was likely favoured by breeding systems where apomixis is facultative and possibly coupled with triploid self-incompatibility^9^. This infrequent sexual route for gene exchange among otherwise clonal species provides the raw variation for adaptation^20^, whilst apomixis maintains the new gene combinations and enables sympatric speciation^4^. However, although SI-PA breeding systems may be responsible for diversification in *Sorbus,* for the species concerned the proximity of suitable mates in both space and time may threaten population sustainability by constraining seed production.

In *Sorbus*, many polyploid species have small population sizes and a high degree of endemism, which are both features of conservation priority species^21^. Threats to the persistence of rare *Sorbus* species arise principally from changing land use, browsing by herbivores (which also prevent recruitment) and competition from invasive non-native plant species^16^. Conserving this evolutionarily dynamic group relies on understanding the factors that affect population viability, which include the influence of pollination on seed production. In order to measure the extent to which pollen limitation constrains seed production in a threatened PA species in *Sorbus*, we investigated the pollination and breeding system of the slender whitebeam, *Sorbus subcuneata* Wilmott, which is putatively self-incompatible.

*Sorbus subcuneata* is a rare triploid species that is endemic to nine sites in Devon and Somerset (southwestern United Kingdom), where it co-occurs with six closely related tetraploid congeners and the common diploid *S. aucuparia* L.^16^,^22^. *Sorbus subcuneata* typically occurs at low relative density as an understorey tree, so spatial isolation may be a constraint on seed production. Here, we report an investigation of the breeding system and pollination ecology of *S. subcuneata* in which we determined: (1) the species’ pollination requirements (i.e. compatibility with conspecific and heterospecific pollen); (2) the extent to which seed set is limited by pollination; and (3) the factors that imposed pollination-limited seed set. Specifically, we evaluated whether pollination limitation arose through either temporal isolation imposed by flowering asynchrony between compatible pollination partners or by spatial isolation of females from suitable male pollen donors.

## Results

### Breeding system

We confirmed that triploid *S. subcuneata* is an obligate pseudogamous apomict, because hand-pollinations demonstrated that seed production required pollination (Table 1) and microsatellite analysis revealed that all of the 95 embryos studied had microsatellite phenotypes that were identical to both their maternal tree and the *S. subcuneata* reference samples shown in Table S1, Supplementary Information.

We found that *S. subcuneata* set seed readily through hand-pollination with congeneric *S. admonitor* pollen, with a flower-to-seed conversion rate of 65% (Table 1). In contrast, 93 hand self-pollinations failed to produce even a single seed. We calculated the highest rate of flower-to-seed conversion that is statistically consistent with zero seed in a sample size of 93 as 3%. Hand-pollination with diploid *S. aucuparia* also failed to yield seed and where pollen was removed and excluded, only one seed developed which contained alleles from *S. admonitor,* so was considered a result of pollen contamination.

**Figure 1:**
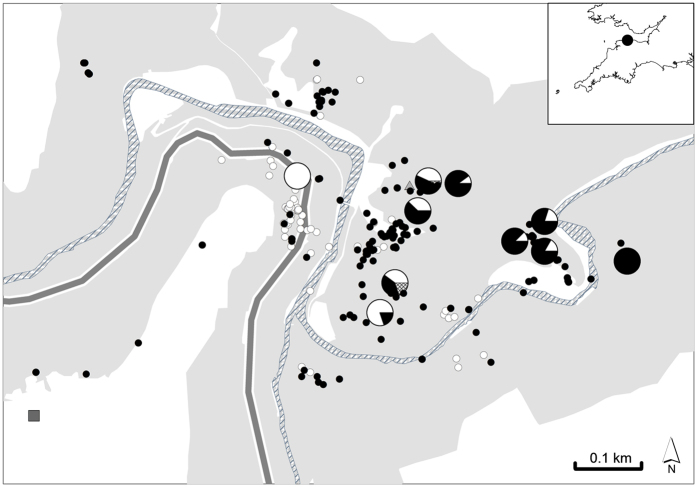
Distribution map of polyploid *Sorbus* species at the woodland site. The locations of *S. subcuneata* maternal seed trees are indicated by pie charts. The sections of the pie charts represent the proportion of seed resulting from pollination (2014 data) by; ⚫ = *S. subcuneata* or ⚬ = *S. admonitor*, 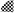 = other. 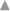 = *S. margaretae*, 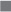 = *S. porrigentiformis.* Survey data supplied by Rich and Cann, (2009) ref. 46. (The map was created using ArcGIS Desktop version 10.2.2, ESRI, California, USA, URL: http://www.esri.com/).

Microsatellite analyses of wild-collected seed showed that endosperm formation resulted from heterospecific pollination by *S. admonitor* in approximately half of the seeds and the other half resulted from self-pollination (Table 2 and Fig. 1). Very rarely (<2% of seeds), the endosperm resulted from pollination by either *S. margaretae*, which is one of the other tetraploid species on the study site (<10 individuals) or diploid *S. aucuparia*.

### Causes of pollen-limited seed set

**Figure 2:**
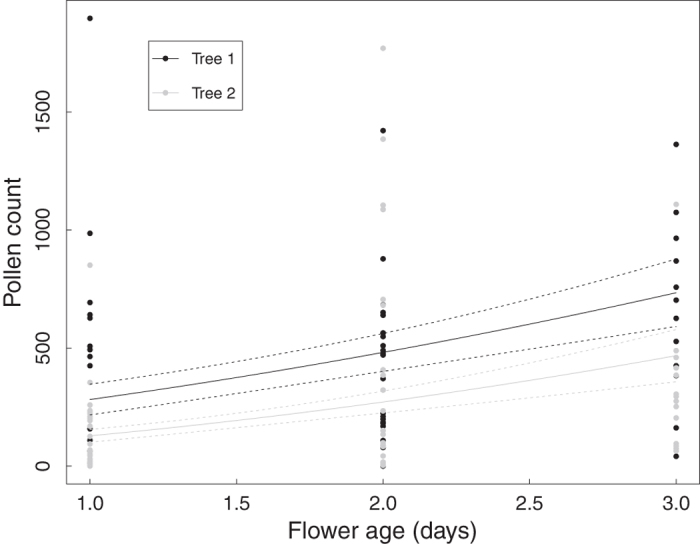
Pollen deposition on the stigmas over the three day life of *S. subcuneata* flowers. Lines show fitted values (±1 SE) from a negative binomial GLM (*square root pollen count* = 11.64 [±2.84] + 5.15 [±1.41] *age - 5.47 [±2.06] *tree, where tree 1 is the reference category). Pseudo R^2^ = 0.102. Tree 1 (upper line): *n* = 58 flowers; tree 2 (lower line): *n* = 68.

Virtually no seed was produced by 1534 open-pollinated *S. subcuneata* flowers on three trees (mean flower to seed conversion rate = 0.5%, SD = 0.46, *n* = 3 trees). Seed production was not limited by the availability of pollen alone because we observed that 88% of two-day old flowers had more than 50 pollen grains on their stigma which had accumulated fairly rapidly over three days (Fig. 2; χ^2^ = 12.11, d.f. = 1, *P* < 0.001). Pollen deposition varied between the two trees studied (χ^2^ = 7.7, d.f. = 1, *P* = 0.005). This pollen accumulation was probably in part due to the activities of the insect pollinators (*Bombus spp., Apis melifera*, Diptera and Lepidoptera) that we observed visiting the flowers.

Seed production in open-pollinated flowers of *S. subcuneata* was strongly limited by the availability of congeneric pollination because supplementation with pollen from *S. admonitor* yielded a flower-to-seed conversion rate of between 12% and 16% (Table 3), whereas those flowers that received no congeneric pollen supplementation yielded no seed.

**Figure 3:**
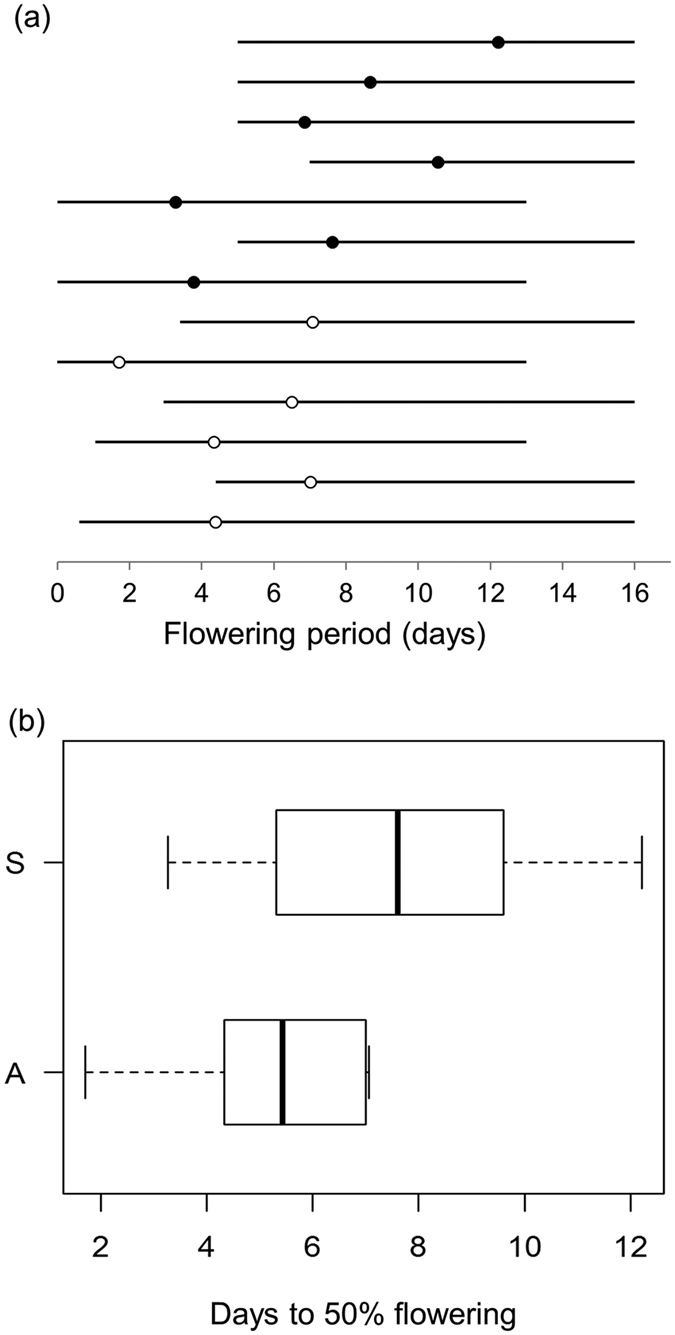
(**a**) Flowering phenology of individual trees of both species over a 16 day flowering period. Each line indicates the flowering period of an individual tree; dots represent the 50 percentile stage of the cumulative flowering curve; *S. subcuneata* (•) *n* = 7 and *S. admonitor* (o) *n* = 6. (**b**) Flowering phenology of both species combined S = *S. subcuneata,* A = *S. admonitor.*

**Figure 4:**
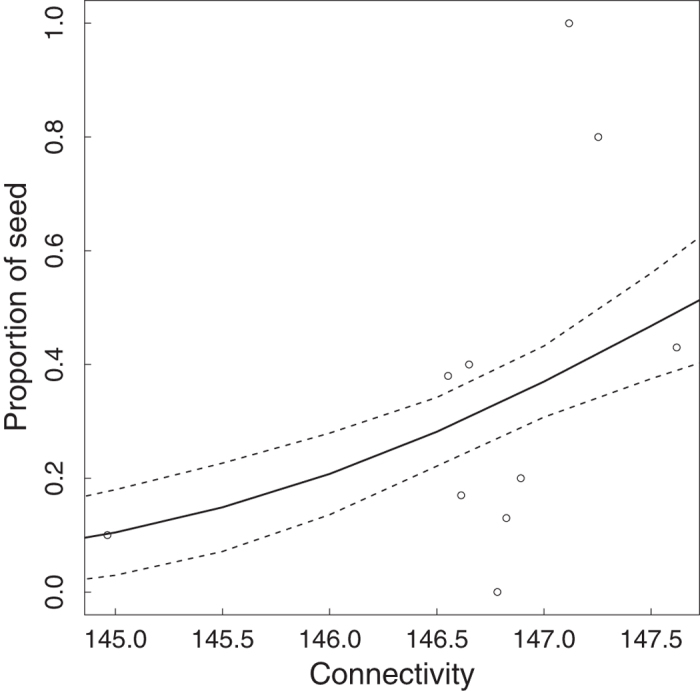
Relationship between the connectivity of the maternal *S. subcuneata* seed trees to *S. admonitor* and the proportion of seed sampled with *S. admonitor* as pollinator (2014 data). The line shows the fitted values (±1SE) for a weighted binomial GLM of: logit (proportion of seed) = −119.1 (±60.1) + 0.81 (±0.41)* *connectivity. N* = 71 seeds from 10 trees.

The lack of congeneric pollination was not the result of asynchrony in blooming of *S. subcuneata* with *S. admonitor*. Pollen flow between these species is certainly possible because the mean flowering day (MFD) was similar for both species (9.4 for *S. admonitor, n* = 6; 11.0 for *S. subcuneata, n* = 7) and there was no significant difference in F_50_ (occasion of 50% of flowers open, Wilcoxon test, W = 12; *P* = 0.23; Fig. 3a and b). Instead, spatial isolation of the maternal trees from compatible pollen donors limited pollen flow between them because the proportion of seed resulting from heterospecific pollination decreased significantly with increasing spatial isolation (i.e. decreasing connectivity) of maternal *S. subcuneata* seed trees from *S. admonitor* individuals (Binomial GLM: connectivity likelihood ratio test, χ^2^ = 4.78, d.f. = 1, *P* = 0.029; pseudo-R^2^ = 0.33; Fig. 4).

Despite the substantially greater efficacy of congeneric pollination in producing seed, male contributions from S*. admonitor* and *S. subcuneata* were represented fairly equally among endosperms of open-pollinated flowers (Table 2) probably because of the relatively high frequency of geitonogamous pollinations. Specifically, based on the relative flower-to-fruit conversion rates we estimate that the ratio of conspecific: heterospecific pollination is *R* = 22:1 (see Tables 1 and 2 and equation 1; *S* = 49.5*, C*_*s*_ = 0.03, *A* = 48.5, and *C*_*a*_ = 0.65).

## Discussion

We found that *S. subcuneata* is normally an obligate apomict. The lack of autonomous endosperm development in our hand pollination control group (no pollen added) confirms *S. subcuneata* as pseudogamous, requiring pollination for successful endosperm formation and seed production in common with other apomictic *Sorbus* spp.^9^,^23^. Thus, the successful pollination of *S. subcuneata* flowers very rarely affects the genotype of the embryo of resulting seed. Our hand pollination experiments showed that the sympatric tetraploid *S. admonitor* is by far the most effective pollinator for *S. subcuneata* on our study site. Indeed, *S. subcuneata* is strongly dependent on its congener because self-pollen is so poorly compatible.

The most likely explanation for the lack of seed produced by conspecific hand pollination is that *S. subcuneata* has a system of gametophytic self-incompatibility (GSI), which has been observed in other triploid *Sorbus* species^9^. GSI is caused by a disruption of the pollen tube before it reaches the ovules and it is controlled by a multi-allelic *S*-locus. GSI occurs when the pollen tube has an *S*-allele in common with the pistil and it is the cause of GSI in diploid *Sorbus* but appears to break down in polyploids with tetraploids exhibiting self-compatibility^24^. High prevalence of inviable pollen is an alternative explanation for the widespread failure to set seed by self-pollination, but this is unlikely because the pollen grains from *S. subcuneata* observed during our study appeared rounded and well-formed and had a higher than average (of triploids tested) stainability^25^, a proxy for pollen viability. We are unable to exclude the possibility that seed failure after selfing is also due to an imbalance between maternal and paternal genome components (m: p) in the endosperm tissue. The 2 m:1p genome ratio of maternal to paternal contributions to the endosperm in sexual species is considered a prerequisite for successful seed development^26^. In pseudogamous apomicts, the lack of meiotic division leads to unreduced central cell polar nuclei in the ovary and fertilisation of these results in m:p ratios that greatly exceed 2 m:1p. However, triploid *Sorbus* such as *S. subcuneata* can tolerate an unbalanced endosperm, which is an attribute shared with other triploid apomicts in the Rosaceae^9^,^27^,^28^. Thus it seems unlikely that the self-incompatibility that we observed in *S. subcuneata* is due to unbalanced endosperm and, instead, the lack of seed production by hand conspecific pollination is likely the result of the operation of GSI.

Despite the greater efficacy of pollination from *S. admonitor* compared to self-pollination, about half of the seeds in open-pollinated flowers arose from conspecific pollination. This finding contrasts to other studies on triploid *Sorbus*^9^ and suggests that the GSI system in *S. subcuneata* is partial, but with a very low rate of success. Self-incompatability can be a quantitive and plastic trait affected by environmental conditions such as temperature at flowering, the composition and density of the pollen load and the internal stylar conditions which change with flower age^29^. A mentor effect whereby a mix of heterospecific and self pollen on the stigma may reduce heterospecific pollination from plants of different ploidy and promote increased selfing in natural mixed ploidy populations^30^. Mentor pollination is widely used as a tool to overcome incompatibility in commercial plant breeding^31^. *S. subcuneata* stigmas pollinated towards the end of the flower life may show reduced self-incompatibility since pollen/pistil interactions during pollen tube growth change with flower age and can allow successful self pollination as opportunities for cross pollination reduce^29^. Whilst these mechanisms may be responsible for a relaxation in self-incompatibility, we cannot rule out the possibility that potentially unsampled genotypes in the *S. subcuneata* population may carry alternative *S* alleles, conferring pollen/pistil compatability and allowing succesfull pollination, although we were unable to find evidence of genetic variation among both seed embryos and adult trees sampled at the study site. The low rate of seed production despite ample pollen deposition in naturally pollinated flowers, however, suggests self-compatibility is a rare phenomenon in *S. subcuneata*.

At our study site, the diploid congener, *S. aucuparia*, was more common than *S. admonitor*, but it was an ineffectual male parent. *S. aucuparia* can successfully pollinate other triploid *Sorbus*^16^,^17^, which shows that compatibility is not determined solely by the comparative ploidy of the parents. The relative efficacy of pollination by tetraploid *S. admonitor* in enabling seed set in closely related triploid *S. subcuneata* probably reflects a broad gametophytic compatibility that permits the successful growth of pollen tubes.

The most striking feature of reproductive ecology in *S. subcuneata* is the low frequency of seed set. At our study site, less than one percent of *S. subcuneata* flowers eventually produced a seed. We observed that open-pollinated flowers of *S. subcuneata* frequently produced parthenocarpic fruits, which suggests that sufficient resources were available for fructification. Further, the low frequency of seed set that we observed was not attributable to the quantity of pollen received by flowers. The flowers of *S. subcuneata* each accumulated an average of 191 grains per day on their stigmas and almost all appeared to be well-pollinated during the three-day duration of anthesis. Normally, the arrival of a minimum or four to six pollen grains per ovule are required for maximum seed set^32^ so there is no evidence that seed set in *S. subcuneata* is limited by the quantity of pollen on its stigmas. Instead, we conclude that the principal limit to seed set is the quality of the pollen that arrives on the stigmas. Specifically, we argue that seed set was limited by the availability of pollen from its congener, S. *admonitor* for two reasons. First, controlled hand-pollination with congeneric pollen greatly increased seed set in otherwise open-pollinated flowers. Second, despite congeneric pollen being more than twenty times more likely than conspecific pollen to initiate seed set, only half of the seeds from successfully open-pollinated flowers contained endosperm with congeneric paternity. Consequently, conspecific pollen must predominate numerically on stigmas over congeneric pollen by the corresponding factor of approximately twenty.

Congeneric pollination from *S. admonitor* was not restricted by asynchronous flowering with *S. subcuneata*, but instead a major result of our study is to show that the availability of congeneric pollen depended on the relative proximity of *Sorbus* congeners.

It is likely that much of the pollen on the stigmas of *S. subcuneata* arrived by insect vectors because we frequently observed bees and flies actively foraging among the entomophilous flowers. Flower-visiting insects tend to make short-distance inter-flower movements when flowers occur in localised patches. Each individual tree of *S. subcuneata* displays hundreds to thousands of flowers simultaneously in anthesis, which enables insects to forage locally and so promotes geitonogamous (within-display) pollen transfer. Consequently, these insects were probably responsible for the preponderance of conspecific pollen on *S. subcuneata* stigmas.

At our site, spatial isolation between congeners is compounded by the locally disjunct distributions of *S. subcuneata* and *S. admonitor* (Fig. 1). Potentially, insect-mediated pollen transfer can span large distances^33^,^34^,^35^, but understorey *Sorbus* individuals receive most of their incoming pollen from neighbours. Our study site is less than two kilometres across and potentially pollen could be transported between any two trees^15^, but the area-restricted movements of flower visitors mean that geitonogamy and short-distance dispersal probably dominated in pollen transfer.

The proportion of seed produced from self-pollination was unrelated to the maternal tree’s proximity to other *S. subcuneata* trees, which further suggests that the large amounts of *Sorbus* pollen that accumulated on the flowers of the maternal trees was principally geitonogamous in origin. Moreover, of *S. subcuneata* and *S. admonitor* on the study site, the closest extrinsic sources of pollen for maternal *S. subcuneata* seed trees were normally other individuals of *S. subcuneata*, not congeners (Fig. 1). Since *S. subcuneata* is effectively clonal, due to its apomictic mode of reproduction, pollen from these conspecific individuals is likely to be as incompatible as that from the maternal tree itself. Together, these attributes could place additional barriers to heterospecific pollination since high levels of geitonogamous pollination will result in increased competition for space on the stigma from unsuitable self-pollen, or ‘stigma clogging’^36^,^37^. Whilst pollen carryover will, to some extent alleviate the effects of geitonogamous pollinator movements^38^, the normally rapid attenuation of carryover^39^,^40^ and localised movements of insect vectors make it inevitable that insect-mediated pollen transfer among large floral displays remains highly restricted in space^41^.

Our study suggests that seed production is likely to be particularly limited in SI-PA species whose individuals are either spatially isolated from compatible congeners or are dispersed in clusters of identical cytotypes. It is likely that our finding applies to some other *Sorbus* triploid species in the UK^42^, which typically exist as small populations sometimes numbering only a few trees^43^. Consequently, effective strategies of conservation management are critical, which we begin to formulate below.

Low rates of seed production such as we have observed make population sustainability particularly precarious in *Sorbus*, which is a palatable species in which recruitment from seeds is threatened by browsing from deer^16^ that exist in large numbers at our study site. When seed production is chronically low, conservation strategies should seek to increase recruitment opportunities to maximise the germination chances of the few seeds currently produced and control herbivore populations. Based on our findings about the reproductive ecology of *S. subcuneata*, however, we urge conservationists also to consider stewardship strategies that encourage close proximity of compatible congeners, which will mitigate pollination-limitation of seed set.

Serendipitously, a strategy that ensures congeneric proximity for ecological reasons is likely also to help to sustain evolutionary diversity in the *Sorbus* lineage. The extremely low reproductive output of *S. subcuneata* in our study population is inherent in its breeding system, which is seemingly maladaptive in a population comprised of a single-cytotype whose individuals have limited access to compatible heterospecific pollen donors. Indeed, although it has been previously predicted on theoretical grounds that SI-PA species should not persist^44^, we have observed a long-established population of c. 300 such trees at our study site. However, although triploids such as *S. subcuneata* may be relatively transient over evolutionary timescales, any capacity for occasional sexual reproduction following congeneric cross-pollination (facultative apomixis) means that they may form important evolutionary stages as part of a recurring process of polyploid formation. The products of facultative apomixis will continually diversify the lineage^45^ because the preponderant involvement of heterospecific pollination in these events increases the frequency of opportunities for hybrid events. In the case of *S. subcuneata*, for example, the high level of allele sharing between *S. subcuneata* and *S. admonitor* suggests recently shared origins from an active process of reticulate evolution by hybridisation. We therefore speculate that a strategy that promotes the proximity of congeners in order to safeguard levels of seed set simultaneously safeguards an evolutionary process in which extinctions of transient microspecies are offset by diversifying hybridisation.

## Materials and Methods

### Study system and sites

We studied a natural population (*c.* 300 trees) of triploid *Sorbus subcuneata* on a woodland site where it occurs with *c*.100 trees of tetraploid *S. admonitor* M.C.F. Proctor and abundant diploid *S. aucuparia*^46^. We focussed our study on these three species as they are all potential pollen sources for *S. subcuneata*, although small numbers of three other tetraploid *Sorbus* species are also found on site: *S. porrigentiformis* E.F. Warb.; *S. margaretae* M.C.F. Proctor and *Sorbus vexans* E.F. Warb. For convenience, pollen compatibility experiments were carried out on two trees held in a collection belonging to the Exmoor Natural History Society (Luckbarrow, West Luccombe, Somerset, UK).

### Investigation of the breeding system of *Sorbus subcuneata*

To determine whether *S. subcuneata* is a SI-PA species, we tested pollen compatibility and whether pollen is required for apomictic seed production (pseudogamy) as follows: (1) pollen added from the same species to test for conspecific compatibility; (2) pollen added from *S. admonitor* (4x); (3) pollen added from *S. aucuparia* (2x); (4) no pollen added to test for autonomous apomixis. All maternal *S. subcuneata* flowers were emasculated on opening and pollen supplementation was conducted 24 hours later to allow stigma maturation. Twenty inflorescences on each of the two maternal trees were randomly assigned to each of the four pollination treatments. We excluded insect pollinators by placing a mesh bag over both the study and pollen donor inflorescences. To reduce the potential effect of resource limitation on seed set, all other inflorescences were removed from the experimental branches. After the application of pollen, the pollen exclusion bags were then replaced for a week to prevent natural pollination. Successful pollination was measured by the production of at least one seed per fruit and calculated as a flower-to-seed conversion rate. Where there was a failure to produce seed in any treatment, we used the binomial theorem to determine the maximum successful conversion rate that would be statistically consistent with this observation at this sample size. The binomial function can be used to calculate *P (x*), the probability of *x* successes in *n* trials. If the probability of success in a single trial is denoted *p*, then *P(x*) is given by:


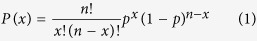


Thus, given the condition that *x* = 0, we solved equation (1) for the maximum value of *p* such that *P(x*) = 0.05, which identified the greatest success rate at which *n* flowers producing zero seed is not statistically significant at the conventional level. In effect, we determined the upper 95% confidence interval on *p.* In addition, we also calculated the overall flower-to-seed conversion rate of naturally pollinated flowers from three trees on the woodland site.

To verify that *S. subcuneata* is an obligate apomict and determine whether interspecific pollen flow contributes to endosperm fertilisation, we used ten nuclear microsatellites previously used for *Sorbus* taxa to genotype the embryo and endosperm of wild-collected, naturally-pollinated seed collected from a total of ten trees in two fruiting seasons (2013 and 2014). Seed embryos produced by apomixis will have a genotype identical to that of the maternal tree. Pollen donors were identified through the presence of their unique alleles in a seed’s endosperm and the proportion of seed resulting from pollination by each congener was calculated. All *Sorbus* species on site were sampled and subjected to genetic analysis to provide reference samples for comparison genotypes. Since polyploid *Sorbus* species are effectively clonal, microsatellite alleles could only be matched to species, not individuals. DNA extraction of leaf and seed material followed QIAGEN DNeasy Plant Minikit protocol. For microsatellite details and PCR conditions see Supplementary Table S2 and Supplementary Methods and references therein. Only samples that successfully amplified across all loci were included in the endosperm paternity analysis with the exception of locus MS14, which only amplified in *S. admonitor, S. subcuneata* and *S. aucuparia.*

To estimate a conspecific: heterospecific pollination ratio of the woodland population we compared the proportion of seed resulting from natural pollination by each congener with the flower-to-seed conversion rates of hand pollinated flowers. The estimated ratio, denoted R, was calculated by:


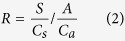


where *S* = total proportion of seed sired by *S. subcuneata, C*_*s*_ = flower-to-seed conversion rate for *S. subcuneata* pollination, *A* = total proportion of seed sired by *S. admonitor*, and *C*_*a*_ = flower-to-seed conversion rate for *S. admonitor* pollination.

### Testing pollen limitation of seed set in the natural population

To investigate whether pollen quantity limited seed production in the woodland population of *S. subcuneata,* we recorded pollen deposition on flower stigmas of two *S. subcuneata* individuals over six days from the onset of flowering until the peak. To determine the amount of pollen on each stigma, a squash preparation was made by softening each sample in sodium hydroxide (8 M NaOH for 10 mins. at 60 °C) before counting the number of pollen grains at x10 magnification. The accumulation of pollen on the flowers of both trees was modelled using a negative binomial generalised linear model (GLM) with square-root link function, which allowed for over-dispersed data. We tested effect of tree and flower age on pollen counts using likelihood ratio tests. This analysis was implemented in the statistical software R^47^.

To investigate whether seed production was limited by pollen quality, we tested whether the application of supplementary compatible pollen increased seed production in open-pollinated flowers of woodland *S. subcuneata*. Based on the outcome of compatibility testing (see Results), we used tetraploid *S. admonitor* as the most favourable male donor. On two maternal trees of *S. subcuneata,* ten inflorescences were selected and each was randomly assigned to one of two treatment groups. Group ‘O’ was left to open pollinate naturally whereas group ‘H’ had heterospecific pollen applied from *S. admonitor.* We assessed the extent of the pollen deficit by comparing the flower-to-seed conversion rate between the H and O treatments.

To investigate whether temporal isolation (asynchronous flowering) could limit pollen flow between maternal trees of *S. subcuneata* and their congeneric pollinator, *S. admonitor*, we quantified the flowering periods of six trees of each species, throughout the 17-day flowering period during May. Every two days, we estimated the proportion of flowers in bud, fully open or in senescence, which was evident because the anthers and petals turned visibly brown. To test for flowering asynchrony between *S. subcuneata* and *S. admonitor*, the relationship of the cumulative proportion of opened buds on each tree over time was fitted by a sigmoid curve, which we used to estimate the time to 50% of flowers open, denoted F_50_. Variation in F_50_ between the two species was analysed using a Wilcoxon Rank-Sum test. For each species, we determined the mean flowering day (MFD), equation (3)^48^, in which % *Fl*_*i*_ is the mean percent of the flowers at anthesis on observation day *i* and *Days*_*i*_ is the number of days elapsed at day_*i*_ from the start of the observation period:


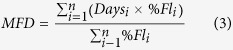


To investigate whether spatial isolation (i.e. spatial separation of maternal trees from compatible pollen donors) could limit seed set in *S. subcuneata*, we used the genetic paternity data (see microsatellite analysis) and correlated the proportion of seed attributable to *S. admonitor* with the maternal tree’s ‘connectivity’ to all potential *S. admonitor* males. The connectivity (*S*_*i*_) for each maternal tree *i* to every potential paternal tree *j* was determined by:


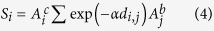


where α modulates the effect of distance between trees *i* and *j*, denoted *d*_*i,j*_, in a negative exponential decay kernel; *A*_*i*_ is the crown size of maternal tree *I*; *A*_*j*_ is the crown size of pollen donor *j*; and *b* and *c* are exponents that scale the impact of tree size on pollen export and import^49^. Connectivity analysis was executed using the SI software^50^. To implement the model, we set α = 0.01 from pollen dispersal patterns measured for *S. torminalis*^15^ and assumed that connectivity was unaffected by the size of the maternal tree because female function is typically satisfied by few pollinator visits^51^ (i.e. *c* = 0) and that male success saturated with increasing tree size (*b* = 0.5)^15^. Distances and crown size were calculated from survey data which had measured location, stem diameter and fruiting for all the polyploid *Sorbus* species on the study site^46^. Crown size (area) was derived from the measurements of diameter at breast height (DBH) using the standard forestry relationship for the architecturally similar congener, *S. aucuparia*^52^. In the analysis, we included only trees of flowering size (trees > 3 cm DBH for *S. admonitor* and >2 cm DBH for *S. subcuneata*).

Using values calculated from equation (4), we tested whether the proportion of seed derived from heterospecific pollen (here *S. admonitor*) produced by each maternal tree (% of total seed) was explained by the connectivity of the maternal seed trees to all *S. admonitor*. To test this hypothesis, we used a GLM with a binomial error and logit link function in which the contribution of seed proportion was weighted by the sample size (number of seeds per tree). This analysis was implemented in the statistical software R^47^. All relevant model assumptions were checked by examining the model deviance residuals and AIC (Akaike information criterion) values were used for model comparisons.

## Additional Information

**How to cite this article:** Hamston, T. J. *et al*. Breeding system and spatial isolation from congeners strongly constrain seed set in an insect-pollinated apomictic tree: *Sorbus subcuneata* (Rosaceae). *Sci. Rep.* **7**, 45122; doi: 10.1038/srep45122 (2017).

**Publisher's note:** Springer Nature remains neutral with regard to jurisdictional claims in published maps and institutional affiliations.

## Supplementary Material

Supplementary Information

## Figures and Tables

**Table 1:** Fruit production at three time intervals after manual pollination with final seed production for four pollination treatments carried out on cultivated specimens of *S. subcuneata*.

Pollen donor	N	Fruit set of *S. subcuneata*						S/F (%)
		July		August		October		
♂		<9 mm	≥9 mm	<9 mm	≥9 mm	Total fruit	Total seed	
*S. admonitor* (4*x*)	**98**	11	46	0	40	40	64	**65.3**
*S. aucuparia* (2*x*)	**92**	13	1	11	0	4	0	**0.0**
*S. subcuneata* (3*x*)	**93**	39	0	8	0	7	0	**0.0**
None	**85**	33	1	10	1	11	1	**1.2**

The two fruit sizes represent small parthenocarpic fruit and larger fruit containing seed at three time intervals after pollination. Pollen donors, with ploidy level in parenthesis, represent the four pollination treatments, N = flowers treated, S/F = percentage of flowers that produced seed.

**Table 2:** Summary of paternity results of microsatellite analysis of seed endosperm from a total of ten maternal *S. subcuneata* trees over two years.

Maternal tree ID No.	Year	N	Paternity (%)		
			*S. subcuneata* (3*x*)	*S. admonitor* (4*x*)	Other
S01	2013	8	13	75	12
S02	2013	3	0	100	0
S269	2013	12	0	100	0
S58	2013	1	0	100	0
S01	2014	14	50	43	7
S02	2014	10	90	10	0
S269	2014	5	20	80	0
S58	2014	1	0	100	0
S156	2014	5	40	60	0
S280	2014	16	63	37	0
S282	2014	1	100	0	0
S283	2014	5	80	20	0
S284	2014	8	88	12	0
S285	2014	6	83	17	0
	TOTAL	95	50 (51)	48 (49)	2

The proportions (%) of seed resulting from the various pollen donor species are shown for each tree in both years. N = number of seed sampled. Proportions in parenthesis are when only seed from *S. subcuneata* and *S. admonitor* are considered, to generate values for equation (1).

**Table 3:** Summary of fruit and seed production for two pollination supplementation treatments carried out on two maternal *S. subcuneata* individuals (S01 and S02).

*S. subcuneata*	Pollen treatment	No. fruit	No. seed	S/F (%)
♀	♂			
S01	H	3	3	**12.0**
S02	H	2	4	**16.0**
S01	O	0	0	**0**
S02	O	0	0	**0**

Each treatment was applied to 25 flowers. H = heterospecific pollen from *S. admonitor* (4*x*), O = naturally open-pollinated, S/F = percentage of flowers that produced seed.
