# Supplementary material for: Breeding system and spatial isolation from congeners strongly constrain seed set in an insect-pollinated apomictic tree: Sorbus subcuneata (Rosaceae)
Source: Sci Rep. 2017 Mar 24;7:45122. doi: 10.1038/srep45122 (PMC5364488; doi:10.1038/srep45122)
Supplement: Supplementary Information [file srep45122-s1.pdf]

Breeding system and spatial isolation from congeners strongly constrain seed set in an insect-pollinated apomictic tree: *Sorbus subcuneata* (Rosaceae)

*Tracey J. Hamston[1, 2], Robert J. Wilson[1], Natasha de Vere[3, 4], Tim C. G. Rich[5], Jamie R. Stevens[1] and James E. Cresswell[1]

[1]Biosciences, University of Exeter, Exeter, Devon, U.K .
[2] Whitley Wildlife Conservation Trust, Paignton Zoo, Paignton, U.K.
[3] National Botanic Garden of Wales, Llanarthne, U.K.
[4] Institute of Biological Environmental and Rural Sciences, Aberystwyth University, Aberystwyth, U.K.
[5] 57, Aberdulais Road, Cardiff, U.K.

*E-mail: thamston@gmail.com

SUPPLEMENTARY INFORMATION

Table S1. Allele sizes for each study *Sorbus* species and seed endosperm from ten maternal *S. subcuneata*. Ploidy levels in parenthesis. Shaded alleles are not present in *S. subcuneata* maternal trees and their presence in seed endosperm indicates heterospecific pollination.

			Microsatellite loci																																							
**Species reference samples**			**MSS16**				**MSS5**				**CH01F09**				**MSS13**				**SA06**				**MS14**				**SA09**				**SA14**				**SA08**				**SA02**			
S. subcuneata (3x)			158	160	204		119	121	123		113	123			193	195			258	268			123				162	194			170		208	226	261				292	294		
S. admonitor (4x)			158	160	188	204	119	121	123	127	113	123			187	193	195		258	268			123	131			162	194			170	178	208	226	261	285			292	294		
S. margaretae (4x)			158	160	162		119	121	135		113	115	123		193	195	197		258	268	280	312	-				162	182	194		194		208	226	247	261			278	282	292	294
S. aucuparia (2x)	sample 262		154				115				-	-			183	193			272	280			125				-	-			212	218			265	267			-	-		
S. aucuparia (2x)	sample 263		154	156			-	-			-	-			199	203			246	264			111	127			-	-			214				257				-	-		
S. aucuparia (2x)	sample 264		154				115				-	-			187	195			272	326			123	125			168	180			214				257				-	-		
S. aucuparia (2x)	sample 507		154				119				-	-			183	187			258				113	123			-	-			214				261				-	-		
**Seed endosperm genotypes**			**MSS16**				**MSS5**				**CH01F09**				**MSS13**				**SA06**				**MS14**				**SA09**				**SA14**				**SA08**				**SA02**			
**Maternal Tree ID**	**Pollen donor**	**Seed ID No.**																																								
**2014 Seed**																																										
S01	*S. admonitor*	1	158	160	204		119	121	123		113	123			187	193	195		258	268			123	131			162	194			170	178	208	226	261				292	294		
S01	*S. admonitor*	2	158	160	204		119	121	123		113	123			187	193	195		258	268			123	131			162	194			170	178	208	226	261	285			292	294		
S01	*S. admonitor*	3	158	160	204		119	121	123		113	123			193	195			258	268			123	131			162	194			170	208	226		261	285			292	294		
S01	*S. admonitor*	4	158	160	204		119	121	123		113	123			193	195			258	268			123				162	194			170	178	208	226	261				292	294		
S01	*S. admonitor*	9	158	160	204		119	121	123		113	123			187	193	195		258	268			123				162	194			170	178	208	226	261	285			292	294		
S01	*S. admonitor*	12	158	160	204		119	121	123		113	123			187	193	195		258	268			123				162	194			170	178	208	226	261	285			292	294		
S01	*S. margaretae*	11	158	160	204		119	121	123		113	115	123		193	195	197		258	268	280	312	123				162	182	194		170	208	226		247	261			282	292	294	
S01	*S. subcuneata*	5	158	160	204		119	121	123		113	123			193	195			258	268			123				162	194			170	208	226		261				292	294		
S01	*S. subcuneata*	6	158	160	204		119	121	123		113	123			193	195			258	268			123				162	194			170	208	226		261				292	294		
S01	*S. subcuneata*	7	158	160	204		119	121	123		113	123			193	195			258	268			123				162	194			170	208	226		261				292	294		
S01	*S. subcuneata*	8	158	160	204		119	121	123		113	123			193	195			258	268			123				162	194			170	208	226		261				292	294		
S01	*S. subcuneata*	13	158	160	204		119	121	123		113	123			193	195			258	268			123				162	194			170	208	226		261				292	294		
S01	*S. subcuneata*	14	158	160	204		119	121	123		113	123			193	195			258	268			123				162	194			170	208	226		261				292	294		
S01	*S. subcuneata*	15	158	160	204		119	121	123		113	123			193	195			258	268			123				162	194			170	208	226		261				292	294		
S156	*S. admonitor*	2	158	160	204		119	121	123		113	123			193	195			258	268			123				162	194			170	178	208	226	261				292	294		
S156	*S. admonitor*	4	158	160	204		119	121	123		113	123			193	195			258	268			123	131			162	194			170	208	226		261				292	294		
S156	*S. admonitor*	3	158	160	204		119	121	123		113	123			193	195			258	268			123				164	194			170	178	208	226	261				292	294		
S156	*S. subcuneata*	1	158	160	204		119	121	123		113	123			193	195			258	268			123				162	194			170	208	226		261				292	294		
S156	*S. subcuneata*	5	158	160	204		119	121	123		113	123			193	195			258	268			123				162	194			170	208	226		261				292	294		
S02	*S. admonitor*	3	158	160	204		119	121	123		111	123			193	195			258	268			123	131			162	194			170	208	226		261	285			292	294		
S02	*S. subcuneata*	2	158	160	204		119	121	123		113	123			193	195			258	268			123				162	194			170	208	226		261				292	294		
S02	*S. subcuneata*	4	158	160	204		119	121	123		113	123			193	195			258	268			123				162	194			170	208	226		261				292	294		
S02	*S. subcuneata*	5	158	160	204		119	121	123		113	123			193	195			258	268			123				162	194			170	208	226		261				292	294		
S02	*S. subcuneata*	6	158	160	204		119	121	123		113	123			193	195			258	268			123				162	194			170	208	226		261				292	294		
S02	*S. subcuneata*	7	158	160	204		119	121	123		113	123			193	195			258	268			123				162	194			170	208	226		261				292	294		
S02	*S. subcuneata*	9	158	160	204		119	121	123		113	123			193	195			258	268			123				162	194			170	208	226		261				292	294		
S02	*S. subcuneata*	11	158	160	204		119	121	123		113	123			193	195			258	268			123				162	164			170	208	226		261				292	294		
S02	*S. subcuneata*	12	158	160	204		119	121	123		113	123			193	195			258	268			123				162	194			170	208	226		261				292	294		
S02	*S. subcuneata*	13	158	160	204		119	121	123		113	123			193	195			258	268			123				162	194			170	208	226		261				292	294		
S269	*S. admonitor*	1	158	160	204		119	121	123		113	123			193	195			258	268			123				162	194			170	208	226		261	285			292	294		
S269	*S. admonitor*	2	158	160	204		119	121	123		113	123			193	195			258	268			123				162	194			170	178	208	226	261				292	294		
S269	*S. admonitor*	3	158	160	204		119	121	123		113	123			187	193	195		258	268			123				162	194			170	208	226		261	285			292	294		
S269	*S. admonitor*	4	158	160	204		119	121	123		113	123			187	193	195		258	268			123	131			162	194			170	208	226		261	285			292	294		
S269	*S. subcuneata*	5	158	160	204		119	121	123		113	123			193	195			258	268			123				162	194			170	208	226		261				292	294		
S280	*S. admonitor*	3	158	160	204		119	121	123		113	123			187	193	195		258	268			123	131			162	194			170	178	208	226	261				292	294		
S280	*S. admonitor*	8	158	160	204		119	121	123		113	123			187	193	195		258	268			123				162	194			170	208	226		261				292	294		
S280	*S. admonitor*	9	158	160	204		119	121	123		113	123			187	193	195		258	268			123				162	194			170	208	226		261	285			292	294		
S280	*S. admonitor*	10	158	160	204		119	121	123		113	123			193	195			258	268			123				162	194			170	178	208	226	261				292	294		
S280	*S. admonitor*	11	158	160	204		119	121	123		113	123			187	193	195		258	268			123				162	194			170	178	208	226	261	285			292	294		
S280	*S. admonitor*	13	158	160	204		119	121	123		113	123			193	195			258	268			123				162	194			170	178	208	226	261				292	294		
S280	*S. subcuneata*	1	158	160	204		119	121	123		113	123			193	195			258	268			123				162	194			170	208	226		261				292	294		
S280	*S. subcuneata*	2	158	160	204		119	121	123		113	123			193	195			258	268			123				162	194			170	208	226		261				292	294		
S280	*S. subcuneata*	4	158	160	204		119	121	123		113	123			193	195			258	268			123				162	194			170	208	226		261				292	294		
S280	*S. subcuneata*	5	158	160	204		119	121	123		113	123			193	195			258	268			123				162	194			170	208	226		261				292	294		
S280	*S. subcuneata*	6	158	160	204		119	121	123		113	123			193	195			258	268			123				162	194			170	208	226		261				292	294		
S280	*S. subcuneata*	7	158	160	204		119	121	123		113	123			193	195			258	268			123				162	194			170	208	226		261				292	294		
S280	*S. subcuneata*	12	158	160	204		119	121	123		113	123			193	195			258	268			123				162	194			170	208	226		261				292	294		
S280	*S. subcuneata*	14	158	160	204		119	121	123		113	123			193	195			258	268			123				162	194			170	208	226		261				292	294		
S280	*S. subcuneata*	16	158	160	204		119	121	123		113	123			193	195			258	268			123				162	194			170	208	226		261				292	294		
S280	*S. subcuneata*	17	158	160	204		119	121	123		113	123			193	195			258	268			123				162	194			170	208	226		261				292	294		
S282	*S. subcuneata*	1	158	160	204		119	121	123		113	123			193	195			258	268			123				162	194			170	208	226		261				292	294		
S283	*S. admonitor*	5	158	160	204		119	121	123		113	123			187	193	195		258	268			123				162	194			170	208	226		261	285			292	294		
S283	*S. subcuneata*	1	158	160	204		119	121	123		113	123			193	195			258	268			123				162	194			170	208	226		261				292	294		
S283	*S. subcuneata*	2	158	160	204		119	121	123		113	123			193	195			258	268			123				162	194			170	208	226		261				292	294		
S283	*S. subcuneata*	3	158	160	204		119	121	123		113	123			193	195			258	268			123				162	194			170	208	226		261				292	294		
S283	*S. subcuneata*	4	158	160	204		119	121	123		113	123			193	195			258	268			123				162	194			170	208	226		261				292	294		
S284	*S. admonitor*	6	158	160	204		119	121	123		113	123			193	195			258	268			123				162	194			170	178	208	226	261	285			292	294		
S284	*S. subcuneata*	1	158	160	204		119	121	123		113	123			193	195			258	268			123				162	194			170	208	226		261				292	294		
S284	*S. subcuneata*	2	158	160	204		119	121	123		113	123			193	195			258	268			123				162	194			170	208	226		261				292	294		
S284	*S. subcuneata*	3	158	160	204		119	121	123		113	123			193	195			258	268			123				162	194			170	208	226		261				292	294		
S284	*S. subcuneata*	4	158	160	204		119	121	123		113	123			193	195			258	268			123				162	194			170	208	226		261				292	294		
S284	*S. subcuneata*	5	158	160	204		119	121	123		113	123			193	195			258	268			123				162	194			170	208	226		261				292	294		
S284	*S. subcuneata*	7	158	160	204		119	121	123		113	123			193	195			258	268			123				162	194			170	208	226		261				292	294		
S284	*S. subcuneata*	8	158	160	204		119	121	123		113	123			193	195			258	268			123				162	194			170	208	226		261				292	294		
S285	*S. admonitor*	5	158	160	204		119	121	123		113	123			193	195			258	268			123				162	194			170	178	208	226	261	285			292	294		
S285	*S. subcuneata*	1	158	160	204		119	121	123		113	123			193	195			258	268			123				162	194			170	208	226		261				292	294		
S285	*S. subcuneata*	2	158	160	204		119	121	123		113	123			193	195			258	268			123				162	194			170	208	226		261				292	294		
S285	*S. subcuneata*	3	158	160	204		119	121	123		113	123			193	195			258	268			123				162	194			170	208	226		261				292	294		
S285	*S. subcuneata*	4	158	160	204		119	121	123		113	123			193	195			258	268			123				162	194			170	208	226		261				294	294		
S285	*S. subcuneata*	6	158	160	204		119	121	123		113	123			193	195			258	268			123				162	194			170	208	226		261				292	294		
S58	*S. admonitor*	1	158	160	204		119	121	123		113	123			193	195			258	268			123				162	194			170	178	208	226	261				292	294		
**2013 Seed**																																										
S01	*S. subcuneata*	0	158	160		204	119	121	123		113	123			193	195			258	268			123				162	194			170		208	226	261				292	294		
S01	*S. admonitor*	1	158	160		204	119	121	123		113	123			193	195			258	268			123				162	194			170		208	226	261	285			292	294		
S01	*S. aucuparia*	6	158	160	152	204	115	119	121	123	113	123			193	195			258	268			111	123			162	194			170	208	214	226	238	261			292	294		
S01	*S. admonitor*	4	158	160	188	204	119	121	123		113	123			193	195			258	268			123	131			162	194			170	178	208	226	261				292	294		
S01	*S. admonitor*	2	158	160		204	119	121	123		113	123			193	195			258	268			123				162	194			170		208	226	261	285			292	294		
S01	*S. admonitor*	5	158	160	188	204	119	121	123	127	113	123			193	195			258	268			123	131			162	194			170	178	208	226	261	285			292	294		
S01	*S. admonitor*	4	158	160		204	119	121	123	127	113	123			193	195			258	268			123	131			162	194			170		208	226	261	285			292	294		
S01	*S. admonitor*	3	158	160	188	204	119	121	123	127	113	123			193	195			258	268			123				162	194			170		208	226	261				292	294		
S02	*S. admonitor*	1	158	160		204	119	121	123		113	123			193	195			258	268			123	131			162	194			170		208	226	261				292	294		
S02	*S. admonitor*	2	158	160		204	119	121	123		113	123			193	195			258	268			123	131			162	194			170	178	208	226	261				292	294		
S02	*S. admonitor*	4	158	160		204	119	121	123	127	113	123			193	195			258	268			123	131			162	194			170	178	208	226	261	285			292	294		
S269	*S. admonitor*	3	158	160		204	119	121	123		113	123			193	195			258	268			123	131			162	194			170	178	208	226	261				292	294		
S269	*S. admonitor*	2	158	160		204	119	121	123		113	123			193	195			258	268			123	131			162	194			170		208	226	261				292	294		
S269	*S. admonitor*	3	158	160		204	119	121	123		113	123			193	195			258	268			123				162	194			170	178	208	226	261	285			292	294		
S269	*S. admonitor*	1	158	160		204	119	121	123		113	123			193	195			258	268			123				162	194			170		208	226	261				292	294		
S269	*S. admonitor*	4	158	160	188	204	119	121	123		113	123			193	195			258	268			123	131			162	194			170		208	226	261	285			292	294		
S269	*S. admonitor*	3	158	160		204	119	121	123		113	123			193	195			258	268			123	131			162	194			170	178	208	226	261				292	294		
S269	*S. admonitor*	3	158	160		204	119	121	123		113	123			193	195			258	268			123	131			162	194			170		208	226	261	285			292	294		
S269	*S. admonitor*	3	158	160	188	204	119	121	123	127	113	123			193	195			258	268			123	131			162	194			170		208	226	261				292	294		
S269	*S. admonitor*	3	158	160	188	204	119	121	123	127	113	123			193	195			258	268			123				162	194			170	178	208	226	261				292	294		
S269	*S. admonitor*	3	158	160		204	119	121	123	127	113	123			193	195			258	268			123	131			162	194			170	178	208	226	261				292	294		
S269	*S. admonitor*	1	158	160		204	119	121	123	127	113	123			193	195			258	268			123				162	194			170		208	226	261				292	294		
S269	*S. admonitor*	3	158	160		204	119	121	123	127	113	123			193	195			258	268			123				162	194			170		208	226	261	285			292	294		
S58	*S. admonitor*	2	158	160		204	119	121	123	127	113	123			193	195			258	268			123				162	194			170		208	226	261	285			292	294		

Table S2. Nucleotide sequences of nuclear microsatellite primers used in this study.

Multiplex	Locus	Primer sequence (5’ to 3’)
MPLX 1	MSS5*	F - CCCCAACAACATTTTTCTCC
		R - CCTCTCGCTCTTTGCCTCT
	MSS16*	F - ATGTCACATCTCTCCCCTTGTGT
		R - TTTTGCCCTCAAAGAATGCCTTA
MPLX 2	CH01F09+	F - ATGTACATCAAAGTGTGGATTG
		R - GGCGCTTTCCAACACATC
	SA06‡	F - ATTTGATCCATGTGCGACTGCA
		R - TGCAGCGGTTGCAGATTGCA
	MSS13*	F - GAAAATTCCTTCCCGAACTTCAT
		R - AACTCACTCGGATTTTGGAACCT
MPLX 3	SA02‡	F - CTAGGTATCATCTCCGACCA
		R - ACGTAGCACTGAATGGTATAG
	SA08‡	F - CAGAGAGAGTGCACTGCCT
		R - GAATTCTTGGCAGTTTGCCT
	SA09‡	F - CTTGTTGGACGGATTTCTTC
		R - CCAATACTTGAGTAGCATAC
	MS14†	F - CGCTCACCATCGTAGACGT
		R - ATGCAATGGCTAAGCATA
Single	SA14‡	F - ATGGATTTAGGTTAACAGTTGTC
		R - GAGGTAAAACCTACCAGTATAC

* Microsatellite primers from *Sorbus torminalis* [1]

+Microsatellite primers derived from *Malus domestica* [2,18]

‡ Microsatellite primers from *Sorbus aria* [3]

† Microsatellite primers derived from *Malus domestica* [4]

Supplementary methods

PCR conditions

The primer pairs were combined into three multiplex reactions and a touchdown polymerase-chain-reaction modified from Hamilton, et al. [5] was carried out in a MyCycler thermal cycler (Bio-Rad, California, USA) according to the following cycling program: 95°C for 5 min; 4 cycles of 95°C for 30 sec, 62°C for 1 min 30 sec and 72°C for 3 min, followed by 4 cycles at decreased annealing temperature of 58°C; 7 cycles at 55°C annealing temperature; 12 cycles at 53°C annealing temperature followed by 3 further sets of 5 cycles at decreasing annealing temperatures in increments of 2°C, and final extension at 72°C for 10 min. The amplified products were analysed using CEQ 8000 Genetic Analysis system (Beckman Coulter, Fullerton, CA, USA).

References

1 Oddou-Muratorio, S. *et al.* Microsatellite primers for *Sorbus torminalis* and related species. *Molecular Ecology Notes* **1,** 297-299, (2001).

2 Gianfranceschi, L., Seglias, N., Tarchini, R., Komjanc, M. & Gessler, C. Simple sequence repeats for the genetic analysis of apple. *Theoretical and Applied Genetics* **96,** 1069-1076, (1998).

3 González-González, E. A., González-Pérez, M. A., Rivero, E. & Sosa, P. A. Isolation and characterization of microsatellite loci in *Sorbus aria* (Rosaceae). *Conservation Genetics Resources* **2,** 341-343, (2010).

4 Nelson-Jones, E., Briggs, D. & Smith, A. The origin of intermediate species of the genus Sorbus. *Theoretical and Applied Genetics* **105,** 953-963, (2002).

5 Hamilton, P. B. *et al.* Populations of a cyprinid fish are self-sustaining despite widespread feminization of males. *BMC biology* **12,** 1, (2014).
